# Characteristics and result reporting of registered COVID-19 clinical trials of Chinese and Indian traditional medicine: A comparative analysis

**DOI:** 10.3389/fmed.2023.1118269

**Published:** 2023-02-17

**Authors:** Nan Zhao, Kritika Pandey, Skanthesh Lakshmanan, Ran Zhao, Jingchun Fan, Junhua Zhang, Max O. Bachmann, Hong Fan, Fujian Song

**Affiliations:** ^1^School of Nursing, Nanjing Medical University, Nanjing, China; ^2^Integrative Ayurveda Network, Aarogyam (UK) CIC, Leicester, United Kingdom; ^3^School of Public Health, Nanjing Medical University, Nanjing, China; ^4^School of Public Health, Gansu University of Traditional Chinese Medicine, Lanzhou, China; ^5^Evidence-Based Medicine Centre, Tianjin University of Traditional Chinese Medicine, Tianjin, China; ^6^Faculty of Medicine and Health Science, Norwich Medical School, University of East Anglia, Norwich, United Kingdom

**Keywords:** COVID-19 clinical trials, China, India, traditional medicine, a comparative analysis

## Abstract

**Objective:**

To assess the main characteristics and result reporting of registered COVID-19 interventional trials of traditional Chinese medicine and traditional Indian medicine.

**Materials and methods:**

We assessed design quality and result reporting of COVID-19 trials of traditional Chinese medicine (TCM) and traditional Indian medicine (TIM) registered before 10 February 2021, respectively, on Chinese Clinical Trial Registry (ChiCTR) and Clinical Trial Registry-India (CTRI). Comparison groups included registered COVID-19 trials of conventional medicine conducted in China (WMC), India (WMI), and in other countries (WMO). Cox regression analysis was used to assess the association between time from trial onset to result reporting and trial characteristics.

**Results:**

The proportion of COVID-19 trials investigating traditional medicine was 33.7% (130/386) among trials registered on ChiCTR, and 58.6% (266/454) on CTRI. Planned sample sizes were mostly small in all COVID-19 trials (median 100, IQR: 50–200). The proportion of trials that were randomized was 75.4 and 64.8%, respectively, for the TCM and TIM trials. Blinding measures were used in 6.2% of the TCM trials, and 23.6% of the TIM trials. Cox regression analysis revealed that planned COVID-19 clinical trials of traditional medicine were less likely to have results reported than trials of conventional medicine (hazard ratio 0.713, 95% confidence interval: 0.541–0.939; *p* = 0.0162).

**Conclusion:**

There were considerable between-country and within-country differences in design quality, target sample size, trial participants, and reporting of trial results. Registered COVID-19 clinical trials of traditional medicine were less likely to report results than trials of conventional medicine.

## Summary box

### What is already known on this topic

• Many clinical trials were registered to evaluate traditional Chinese and Indian remedies for the treatment of COVID-19.
• Previous studies have characterized registered COVID-19 clinical trials of traditional medicine. However, there were no studies on result reporting of such registered trials.

### What this study adds

• There were considerable between-country and within-country differences in design quality, target sample size, types of trial participants, and reporting of trial results.
• Registered COVID-19 clinical trials of traditional medicine were less likely to report results than trials of conventional medicine.

### How this study might affect research, practice or policy

• Lessons learned from COVID-19 clinical trials of traditional medicine will contribute to the improvement of the design, conduct, and reporting of clinical trials of traditional medicine for the ongoing COVID-19 pandemic and new emerging acute infectious diseases in future.

## Introduction

Coronavirus disease 2019 (COVID-19) caused by severe acute respiratory syndrome coronavirus 2 (SARS-CoV-2) was first reported in December 2019, and the World Health Organization (WHO) declared the COVID-19 a global pandemic on 11 March 2020 (1). Because of no proven conventional treatments for COVID-19 at the early phase of the pandemic, traditional remedies were adopted in many countries, including the most populous countries of the world, China and India (2–4). The use of traditional medicine for COVID-19 was initially based on previous experience of the treatment of similar symptoms and respiratory diseases (5). Simultaneously, many COVID-19 clinical trials of traditional medicine were registered since the onset of COVID-19 outbreak (6, 7).

Serious concerns were raised regarding the registered COVID-19 clinical trials, including waste of research sources due to poor quality, small sample sizes, redundancy, or unnecessary duplication (8–10). Although previous studies have characterized registered COVID-19 clinical trials of traditional medicine, we are not aware of studies on result reporting of such registered trials. To avoid the waste of research resources, results of all clinical trials should be reported, including results of underpowered studies and so-called failed clinical trials with negative results, which may contribute to evidence-based medicine and the design of further studies (11). This study aimed to assess and compare the main characteristics and result reporting of registered COVID-19 clinical trials of traditional Chinese and Indian medicine, and to reveal lessons to be learned from COVID-19 clinical trials of traditional medicine.

## Materials and methods

### Inclusion/exclusion criteria

A database of COVID-19 clinical trials registered in multiple trial registries globally is available from the International Clinical Trials Registry Platform (ICTRP) of the World Health Organization (WHO).^1^ Relevant trials were identified from the WHO ICTRP database of registered COVID-19 clinical trials downloaded on 10 February 2021.

We included COVID-19 trials of traditional Chinese medicine (TCM) registered on Chinese Clinical Trial Registry (ChiCTR), and COVID-19 trials of traditional Indian medicine (TIM) registered on Clinical Trials Registry -India (CTRI). For making comparisons between trials of traditional and conventional medicine, and between countries, we also included COVID-19 clinical trials of conventional (or Western) medicine registered on ChiCTR, CTRI, and a random sample of 400 trials registered on Clinical.Trials.gov. Microsoft Excel (for Microsoft 365 MSO) was used to manage trials registered on ChiCTR, CTRI and Clinical.Trials.gov. We used Excel RAND command to generate a random number (from 0 to 1) for each of trials registered on Clinical.Trials.gov, and selected the first 400 after ordering by the assigned random number from the smallest to the largest. We excluded observational or other non-interventional studies, duplicate registry entries, trials registered on ChiCTR but conducted in countries other than China, and trials registered on CTRI but conducted in countries other than India.

### Data extraction

Data extraction and searching of reported trials were conducted independently by two reviewers, and any disagreements between the reviewers were resolved by discussion. The ICTRP COVID-19 database contains information on trial registration number (TRN), country, design, target sample size, trial sponsors, interventions evaluated, inclusion and exclusion criteria, date of registration, recruitment status, date of trial onset, and so on. We checked the original trial registration if the required data were missing or unclear in the ICTRP database. We categorized trial primary sponsors as industry or non-industry. Trials were categorized as randomised controlled trial (RCT) or not, parallel or not, and with any blinding measures or without. Evaluated interventions were categorized as: pharmacological, alternative/dietary, immunological (including antibody and convalescent plasma), vaccine, stem cell, digital health, ventilation/oxygen, physical therapy/rehabilitation, and behavioral/psychological. These intervention categories may not be mutually exclusive. Alternative/dietary interventions included traditional medicine, other alternative or complementary remedies, and dietary supplementary interventions. Traditional Chinese medicine (TCM) included herbal, compound formulas, acupuncture, and other traditional remedies. Traditional Indian medicine (TIM) included Ayurveda, yoga, naturopathy, Unani, Siddha, and homeopathy. Registered trials were categorized as trials including patients with severe, non-severe and recovered COVID-19 cases, and individuals without COVID-19 at enrolment. “Non-severe” cases included patients from asymptomatic, mild or moderate severity, but trials that included cases from moderate to severe or critical severity were categorized as trials of severe cases. Participants without COVID-19 at enrolment included healthy volunteers, health workers, contacts of confirmed cases, or general community residents.

To identify trials that reported results, we first checked fields in the trial registers regarding result reporting and publications. If there was no information on result reporting in the register, we searched Google or Microsoft Bing using the unique trial registration number (TRN) as search term. Any reporting of outcome results was eligible, including interim results before trials’ completion. Types of result reporting were categorized as preprint, journal article, result posted on trial register, and news release. If the result of a clinical trial was reported through multiple approaches, we used the date and type of the earliest open access reporting.

### Data analysis

We compared characteristics of registered COVID-19 clinical trials between traditional medicine and conventional medicine within and across countries. To compare characteristics of different groups of trials, we used Pearson’s chi-squared test (with simulated *p*-values in cases of small event numbers) for categorical variables, and used non-parametric Kruskal-Wallis rank sum test for continuous variables that were not normally distributed. Statistical significance was defined as two-sided *P* < 0.05.

The primary endpoint is time from trial onset (start of enrolment) to the first reporting of results. We conducted survival analysis to compare time (days) from trial onset to the first reporting of results using Kaplan-Meier survival curves and the log rank test. Time from onset to result reporting was censored on 20 April 2022, which was also applied to registered trials that never started or were early terminated, and trials that reported results after 20 April 2022. We conducted Cox proportional hazards regression analysis to assess the association between time from trial onset to result reporting and trial characteristics (including traditional or conventional medicine, country, industry-sponsor, randomized or not, sample size, and dates of registration). We used R computing language for statistical analyses, (12) and R “survival” package for Cox proportional hazards regression models (13).

### Patient and public involvement

Patient and public were not directly involved in this study.

## Results

### The main characteristics of the registered trials

**FIGURE 1:**
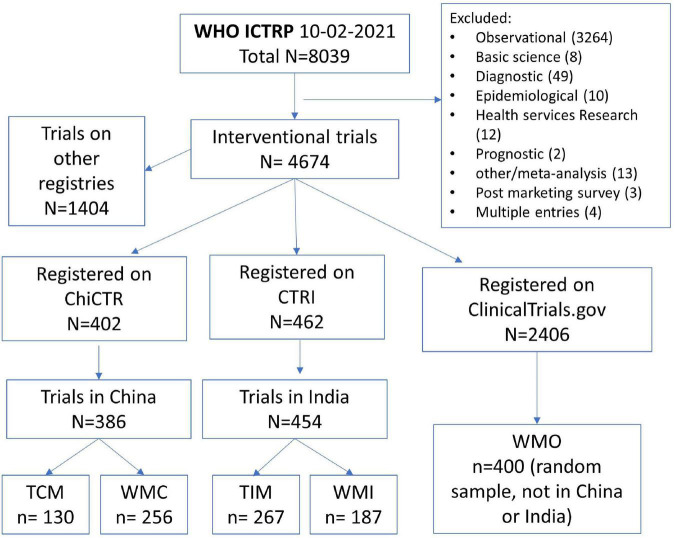
Process of selecting registered COVID-19 interventional trials. ChiCTR, Chinese Clinical trial Registry; CTRI, Clinical Trials Registry-India; TCM, traditional Chinese medicine; WMC, trials on conventional (western) medicine in China; TIM, traditional Indian medicine; WMI, trials on conventional medicine in India; WMO, conventional or other medicine in countries other than China and India.

The process of selection of registered COVID-19 trials is shown in Figure 1. We finally included 130 registered COVID-19 clinical trials of traditional Chinese medicine (TCM), 256 trials of conventional medicine in China (WMC), 267 trials of traditional Indian medicine (TIM), 187 trials of conventional medicine in India (WMI), and a random sample of 400 trials conducted in other countries (WMO). The proportion of trials of traditional medicine was 33.7% among the trials registered on ChiCTR, and 58.8% among the trials registered on CTRI. The main characteristics of the included trials are shown in Table 1.

**TABLE 1:** The main characteristics of registered COVID-19 clinical trials included in the study.

Characteristic	Traditional Chinese medicine trials N (%)	Western medicine trials in China N (%)	Traditional Indian medicine trials N (%)	Western medicine trials in India N (%)	COVID-19 trials in other countries N (%)
**Total**	130 (100.0%)	256 (100.0%)	267 (100.0%)	187 (100.0%)	400 (100.0%)
**Date of registration:**					
Median (IQR), day/month/year	24/2/20 (13/2/20, 17/3/20)	3/3/20 (18/2/20, 5/4/20)	16/7/20 (5/6/20, 15/9/20)	10/8/20 (6/6/20, 30/10/20)	21/6/20 (27/4/20, 9/9/20)
Prospectively registered	74 (56.9%)	139 (54.3%)	267 (100.0%)	187 (100.0%)	396 (99.0%)
**Country:**					
China	130 (100.0%)	256 (100.0%)	0 (0.0%)	0 (0.0%)	0 (0.0%)
India	0 (0.0%)	0 (0.0%)	267 (100.0%)	187 (100.0%)	0 (0.0%)
USA	0 (0.0%)	0 (0.0%)	0 (0.0%)	0 (0.0%)	126 (31.5%)
Multiple	0 (0.0%)	0 (0.0%)	0 (0.0%)	0 (0.0%)	40 (10.0%)
Other	0 (0.0%)	0 (0.0%)	0 (0.0%)	0 (0.0%)	234 (58.5%)
**Recruitment status:**					
Completed	31 (23.8%)	54 (21.1%)	74 (27.7%)	57 (30.5%)	215 (53.8%)
Not recruiting	44 (33.8%)	87 (34.0%)	158 (59.2%)	110 (58.8%)	50 (12.5%)
Recruiting	49 (37.7%)	90 (35.2%)	34 (12.7%)	18 (9.6%)	100 (25.0%)
Terminated	6 (4.6%)	25 (9.8%)	1 (0.4%)	2 (1.1%)	35 (8.8%)
**Trial primary sponsor:**					
Industry	0 (0.0%)	7 (2.7%)	58 (21.7%)	44 (23.5%)	110 (27.5%)
Non-industry	130 (100.0%)	249 (97.3%)	209 (78.3%)	143 (76.5%)	290 (72.5%)
**Trial design:**					
Randomized	98 (75.4%)	179 (69.9%)	173 (64.8%)	136 (72.7%)	324 (81.0%)
Blinded	8 (6.2%)	35 (13.7%)	63 (23.6%)	53 (28.3%)	203 (50.8%)
Parallel	102 (78.5%)	202 (78.9%)	174 (65.2%)	136 (72.7%)	334 (83.5%)
**Study phase:**					
Phase 0, not applicable, unknown	105 (80.8%)	172 (67.2%)	83 (31.1%)	88 (47.1%)	94 (23.5%)
Phase 1 or 1/2	3 (2.3%)	18 (7.0%)	14 (5.2%)	10 (5.3%)	66 (16.5%)
Phase 2 or 2/3	0 (0.0%)	1 (0.4%)	98 (36.7%)	39 (20.9%)	157 (39.3%)
Phase 3 or 3/4	0 (0.0%)	15 (5.9%)	64 (24.0%)	44 (23.5%)	65 (16.3%)
Phase 4	22 (16.9%)	50 (19.5%)	8 (3.0%)	6 (3.2%)	18 (4.5%)
**Target sample size:**					
≤ 50	16 (12.3%)	77 (30.1%)	57 (21.3%)	61 (32.6%)	119 (29.8%)
51–100	34 (26.2%)	80 (31.3%)	85 (31.8%)	53 (28.3%)	89 (22.3%)
101–200	47 (36.2%)	46 (18.0%)	66 (24.7%)	26 (13.6%)	66 (16.5%)
201–400	23 (17.7%)	35 (13.7%)	15 (5.6%)	19 (10.2%)	51 (12.5%)
> 400	10 (7.7%)	17 (6.6%)	44 (16.5%)	27 (14.4%)	60 (15.0%)
**Trial participants:**					
Severe	13 (10.0%)	55 (21.5%)	7 (2.6%)	40 (21.4%)	100 (25.0%)
Mixed cases	49 (37.7%)	118 (46.1%)	36 (13.5%)	45 (24.1%)	172 (43.0%)
Non-severe	29 (22.3%)	30 (11.7%)	142 (53.2%)	52 (27.8%)	47 (11.8%)
Recovery	27 (20.8%)	4 (1.6%)	1 (0.4%)	2 (1.1%)	4 (1.0%)
Other (without COVID-19)	12 (9.2%)	49 (19.1%)	81 (30.3%)	48 (25.7%)	77 (19.3%)
Interventions:*					
Pharmacological	12 (9.2%)	116 (45.3%)	5 (1.9%)	88 (47.1%)	231 (57.8%)
Alternative	130 (100.0%)	11 (4.3%)	267 (100.0%)	6 (3.2%)	26 (6.5%)
Immunological	8 (6.2%)	35 (13.7%)	1 (0.4%)	38 (20.3%)	77 (19.3%)
Physical, rehabilitation	8 (6.2%)	17 (6.6%)	5 (1.9%)	12 (6.4%)	17 (4.3%)
Vaccine	0 (0.0%)	21 (8.2%)	1 (0.4%)	6 (3.2%)	26 (6.5%)
Behavioral, psychological	4 (3.1%)	25 (9.8%)	2 (0.7%)	4 (2.1%)	17 (4.3%)
Digital health technology	1 (0.8%)	12 (4.7%)	2 (0.7%)	11 (5.9%)	19 (4.8%)
Ventilation/Oxygen	0 (0.0%)	7 (2.7%)	0 (0.0%)	19 (10.2%)	20 (5.0%)
Stem cell	0 (0.0%)	24 (9.4%)	0 (0.0%)	2 (1.1%)	13 (3.3%)
Other	0 (0.0%)	25 (9.8%)	0 (0.0%)	18 (9.6%)	37 (9.3%)

*Multiple interventions were evaluated in some registered trials.

The proportion of trials registered prospectively was 55.2% among trials on ChiCTR, compared with 100 and 99%, respectively, among trials on CTRI and Clinical.Trials.gov (Table 1). The proportion of industry-sponsored trials was much lower in China (1.8%), compared with India and other countries (24.8%). The median date of trial registration was 28 February 2020 for trials in China, 20 July 2020 in India, and 21 June 2020 in other countries (Table 1). The distribution of dates of trial registration corresponded to the first wave of the COVID-19 outbreak in different countries (Supplementary Figure 1). By 20 April 2022, the proportion of registered trials that never started, withdrew, or were terminated early was 8.0, 0.7, and 8.8%, respectively, for trials in China, India, and other countries. Reasons given for not starting or early terminating registered trials included mainly difficulty recruiting participants after suppression of COVID-19 outbreaks, availability of new evidence from other research, and possible harmful effects of treatments evaluated.

The proportion of trials of traditional and dietary interventions was 36.5, 60.1, and 6.5%, respectively, among registered trials in China, India, and other countries. Participants targeted by trials of traditional medicine tended to be different from trials of conventional medicine. Non-severe COVID-19 cases were recruited in 22.3% of the TCM trials and in 53.2% of the TIM trials, compared with 11.7% of the WMC trials and 27.8% of the WMI trials. Relatively more TCM trials focused on severe COVID-19 cases (10.0%) than TIM trials did (2.6%). Participants recruited without COVID-19 were in 9.2% of the TCM trials, compared with 30.3% of the TIM trials. Furthermore, 20.8% of TCM trials targeted on patients who were recovering or recovered from COVID-19, which was the highest among trial groups (Supplementary Figure 2).

Planned sample sizes were small in most registered COVID-19 trials [median 100, interquartile range (IQR) 50–200]. The proportion of trials with a target sample size > 400 was only 7.0% in trials registered on ChiCTR, which was lower than trials registered on CTRI (15.6%) and Clinical.Trials.gov (15.0%) (Table 1). Target sample sizes of registered trials were statistically significantly associated with types of participants (Supplementary Figure 3). Small trials were more likely to recruit severe COVID-19 cases, and large trials were more likely to include healthy volunteers or other individuals without COVID-19.

Randomised controlled trials (RCTs) accounted for 75.4, 69.9, 64.8, 72.7, and 81.0%, respectively, among the TCM, WMC, TIM, WMI, and WMO trials. The registered TCM trials were less likely to use any blinding measures (6.2%), compared with WMC trials (13.7%), TIM trials (23.6%), WMI trials (28.3%), and WMO trials (50.8%). The proportion of registered trials with missing or irrelevant study phase information was 80.8% for TCM trials, much higher than in other trials groups. However, 18.7% of registered trials in China were stated to be phase 4, which was much higher than trials in India (3.1%) and other countries (4.5%).

### Trial result reporting

There were statistically significant differences (*P* < 0.001) in the format of result reporting by trial group (Table 2). Most results were reported as journal articles for TCM trials (86.7%), which was higher than 66.7, 68.5, 68.0, and 49.0%, respectively, for the WMC, TIM, WMI, and WMO trials. Reporting as preprints was lowest for the TCM trials (10.0%) and highest for the WMI trials (32.0%). Of the 34 trials that firstly reported results in trial registers, 33 were reported on Clinical.Trials.gov, and one on ChiCTR.

**TABLE 2:** Types of result reporting of registered COVID-19 clinical trials.

Characteristic	Traditional Chinese medicine (130)	Western medicine–China (256)	Traditional Indian medicine (267)	Western medicine–India (187)	Western medicine–other countries (400)
Reported trials	30 (100%)	60 (100%)	54 (100%)	50 (100%)	145 (100%)
**Type of reporting**					
Journal	26 (86.7%)	40 (66.7%)	37 (68.5%)	34 (68.0%)	71 (49.0%)
Preprint	3 (10.0%)	18 (30.0%)	14 (25.9%)	16 (32.0%)	37 (25.5%)
Registry	0 (0.0%)	1 (1.7%)	0 (0.0%)	0 (0.0%)	33 (22.8%)
News	1 (3.3%)	1 (1.7%)	3 (5.6%)	0 (0.0%)	4 (2.8%)

Differences in reporting types across trial groups were statistically significant: Pearson’s Chi-squared test with simulated *p*-value (based on 3,000 replicates): X-squared = 56.248, df = NA, *p*-value = 0.0003332.

**FIGURE 2:**
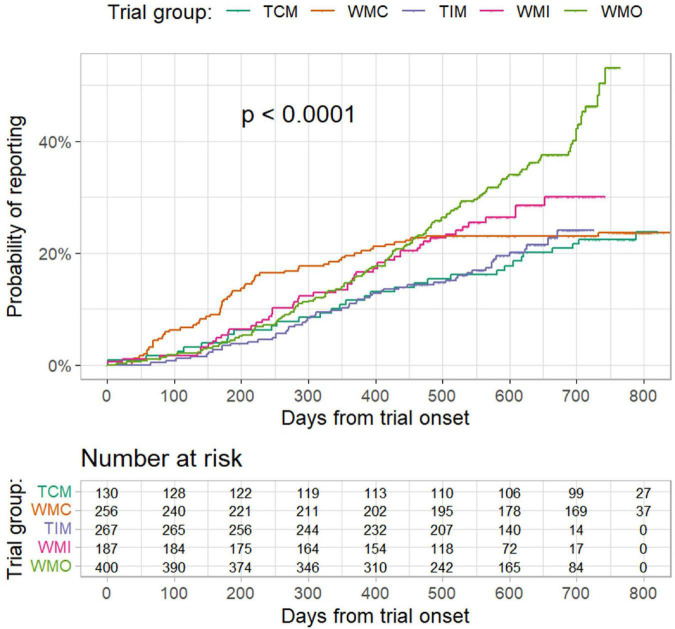
Probability of result reporting from trial onset to 20 April 2022 by different trial groups. TCM refers to “traditional Chinese medicine”; WMC refers to “trials of conventional medicine in China”; TIM refers to “trials of traditional Indian medicine”; WMI refers to “trials of conventional medicine in India”; and WMO refers to “trials conducted in other countries”. *P* values were from log-rank test.

The proportion of trials reported results at 6 months from trial onset was 5.4% (95% CI: 1.4–9.2%), 12.5% (8.4–16.5%), 3.4% (1.2–5.5%), 6.4% (2.8–9.9%), and 4.3% (2.3–6.3%), respectively, for the TCM, WMC, TIM, WMI, and WMO trials (Figure 2). According to results of Cox regression analysis (Table 3), planned COVID-19 clinical trials of traditional medicine were less likely to have results reported than trials of conventional medicine (hazard ratio: 0.713, 95% confidence interval (CI): 0.541–0.939; *p* = 0.0162). Furthermore, trials conducted in other countries, sample size > 100, industry-sponsored, and earlier registered trials were more likely to have results reported (Table 3).

**TABLE 3:** Results of cox multiple regression analysis of result reporting.

Variables	Hazard ratio	(95%CI)	*P*-value
Traditional/Dietary	0.713	(0.541–0.939)	0.0162
Country: China	0.621	(0.464–0.832)	0.0014
Country: India	0.700	(0.525–0.934)	0.0154
Randomized	1.069	(0.827–1.383)	0.6091
Severe cases	0.863	(0.647–1.151)	0.3165
Size *n* > 100	1.319	(1.063–1.638)	0.0121
Registered earlier	1.350	(1.082–1.685)	0.0080
Industry sponsored	1.515	(1.159–1.979)	0.0023

Hazard ratio HR > 1 indicated that a variable was associated with a higher rate of result reporting than the reference. 95% CI refers to 95% confidence interval. “Traditional” medicine included alternative and dietary interventions. Reference groups were the trials of conducted in other countries, not randomized, any trials in which participants were not categorized as “severe,” sample size ≤ 100, and registered after the median date of trial registration. Likelihood ratio test = 57.49 on 8 df, *p* = 1.446e-09, *n* = 1,223, number of events = 338 (17 observations deleted due to missingness).

Of the trials that reported results, the median days from trial onset to result reporting was 350.0 days (IQR 200.0–483.0), and the differences in days from trial onset to result reporting were statistically significant (*P* < 0.001) between the groups of trials (Supplementary Figure 4). The elapse from trial onset to result reporting was shortest for the WMC trials (median days 176.5; IQR 91.3–295.3).

## Discussion

Trials of traditional medicine accounted for about a third of COVID-19 clinical trials registered on ChiCTR and more than half of COVID-19 trials registered on CTRI. Only 55% of the COVID-19 trials in China were prospectively registered, which might be due to the perceived need to activate clinical trials urgently or lack of research preparedness in the early phase of the COVID-19 outbreak. The temporal distribution of newly registered COVID-19 clinical trials corresponded with the geographical location of the initial COVID-19 outbreaks, starting earlier in China than in India and other countries. After suppression of the first wave of COVID-19 outbreak by effective lockdown measures, many registered COVID-19 clinical trials never started or were prematurely terminated.

The target sample sizes of registered COVID-19 trials were small in general. There were fewer registered trials with a target sample size > 400 in China, compared with trials in India and other countries. Planned sample sizes were associated with types of trial participants. Severe COVID-19 cases were more likely to be included in small trials, and healthy volunteers or other individuals without COVID-19 were more likely included in large trials. Traditional Chinese remedies were more likely to be used in severe and recovering COVID-19 cases, and traditional Indian remedies were more likely to be used in non-severe cases and for the preventative purpose among individuals without COVID-19. Regarding design quality, the majority of planned COVID-19 trials were RCTs. However, blinding methods were used in only 6.2% of the trials of traditional Chinese medicine, compared with 23.7% among the trials of traditional Indian medicine. Lack of blinding may bias trial results, particularly for short-term and subjectively measured outcomes. It is noticeable that the proportion of registered phase-4 trials in China (18.7%) was much higher than that in India (3.1%) and other countries (4.5%).

We found that the cumulative probability of result reporting of registered COVID-19 trials in other countries (36.3%) was higher than trials in China (23.3%) and in India (22.9%). In a previous study of 516 COVID-19 RCTs registered before 10 April 2020, 10.3% reported results by October 2020 (14). Another study found that, among 285 completed COVID-19 clinical trials, 18.9% reported results within 3 months after trial completion (15). Mayer and Huser reported that 17.8% of 3,167 COVID-19 trials registered on ClinicalTrials.gov had at least one linked result article by 31 January 2022 (16). The proportion of result reporting in registered COVID-19 trials in our study tended to be higher than the estimated in other studies. However, our results may not be comparable with findings from these studies, due to different inclusion criteria, dates of trial registration, and time of following up from trial onset or completion.

To meet the urgent needs for research information and avoid delays in publishing trial results, the extensive use of open access preprints for rapid research dissemination is unprecedented (17). The overall proportion of preprints in the reported trials was 26.0% in this study, which was similar to the 24.5% from a previous study, (15) and lower than the 51.2% from another study (14). Trials of traditional Chinese medicine were less likely to be reported as preprints (10.0%), compared with other trial groups. A considerable proportion of results of COVID-19 trials registered on Clinical.Trials.gov were initially posted in trial registers (22.8%), while such reporting was rare for trials registered on ChiCTR and CTRI. Early posting of results on trial registers to reduce delays of research dissemination should be encouraged, although further study is required to understand the discrepancies between the early posted results in trial registries and subsequent peer-reviewed journal articles.

### Potential role of traditional medicine

Traditional remedies have been emphasized as a response to the COVID-19 outbreak in many countries, due to no proven treatments initially, historical experiences, their general availability, and national endorsing policies (2). An overview of 23 systematic reviews found that traditional Chinese medicine combined with Western medicine may be beneficial for COVID-19 in term of a wide range of clinical outcomes (18). Evidence provided at a WHO expert meeting indicated that TCM treatments were beneficial for non-severe COVID-19 cases, although data on severe cases was limited (19). The potential benefits of TCM treatments may result from their direct antiviral activities but, arguably more likely, by balancing the immune system and alleviating the harmful cytokine storm (20, 21). These speculated mechanisms prompted relatively more TCM trials that included severe cases and recovering patients. Similarly, basic research and available results of some clinical trials indicated potential antiviral or immunomodulatory properties, and therapeutic usefulness, of Ayurvedic formulations (6, 22). We found that registered trials of traditional Indian medicine were more likely to be targeted on non-severe COVID-19 cases or individuals without COVID-19 illness, which might be due to the perceived preventative mechanisms of Ayurvedic formulations. Findings from computer simulation (*in silico*) studies indicated that phytoconstituents from Ayurvedic formulations might inhibit the entry of coronaviruses into host cells, by having high binding affinity to angiotensin converting enzyme 2 (ACE2) and SARS-CoV-2 S protein (6).

### Lessons learnt and implication for future research

Because there were no proven treatments at the early phase of the pandemic, healthcare professionals relied on previous experience, repurposed interventions, and anecdotes when making decisions about COVID-19 prevention and treatment (23). Simultaneously, a large number of planned clinical trials were registered swiftly, which indicated that healthcare professionals, including traditional medicine professionals, recognized that the treatments they were using should be properly evaluated in clinical trials. This can be considered as an achievement of evidence-based medicine during past decades. However, results of the current and other studies indicated that registered COVID-19 clinical trials on traditional medicine were often underpowered, poorly designed, and unlikely to provide valid and relevant evidence (24–27). A large proportion of the COVID-19 clinical trials in China was retrospectively registered, possibly due to the perceived urgency to activate planned clinical trials or inadequate awareness of the importance of prospective trial registration. The results of many COVID-19 trials may be biased because of inadequate trial design.

It is often challenging to conduct clinical trials as a response to emerging acute infectious diseases like COVID-19, as the outbreak may emerge abruptly and often end quickly because of non-pharmaceutical measures, the development of effective vaccines and widespread immunity. Many registered COVID-19 trials never started or were terminated early, due to the successful suppression of the initial COVID-19 outbreak in China. This fact should be taken into consideration in the design of clinical trials when new acute infectious diseases emerge in future. There have been some successful COVID-19 clinical trials, such as the RECOVERY platform trial in the UK, (28) which provide helpful experiences to inform the design of clinical trials of traditional medicine for emerging infectious diseases in future (10).

In general, the registered COVID-19 trials of traditional medicine less likely reported results than the registered trials of conventional medicine. The COVID-19 trials started early in China, and results of clinical trials conducted in China were possibly easily accepted for publication given the extreme scarcity of information in the early phase of the pandemic. Of the reported trials, trials of conventional medicine in China had the shortest period from trial onset to result reporting across the trial groups. However, the speed of result reporting of the reported TCM trials was similar to the reported trials in India and other countries.

Traditional remedies were usually based on hundreds or thousands of years of experience. The COVID-19 pandemic provided an opportunity for intensive evaluation of relevant traditional remedies in clinical trials. Results of all trials on traditional medicine, either positive or negative, need to be disseminated in order to contribute to unbiased evidence-based traditional medicine. Traditional medicine treatments proven effective in valid clinical trials should be applied in practice, and negative results of valid clinical trials can help preclude the use of ineffective or harmful interventions. Of the COVID-19 clinical trials registered before 10 February 2021, 30 on TCM and 54 on TIM had reported results by 20 April 2022. Reported COVID-19 trials of traditional medicine have contributed or will contribute to evidence-based medicine in relevant systematic reviews and clinical guidelines.

### Study limitations

Because of resource and expertise restrictions, we focused on registered COVID-19 trials on tradition medicine in China and India, and did not considered those in other countries. We only included COVID-19 trials of traditional medicine registered on ChiCTR and CTRI, even though some TCM and TIM trials were registered on other trial registries. We may have missed some published or otherwise reported results by searching on trial registration number (TRN). We found that the TRN of many studies was not mentioned in abstracts, and was discovered only by checking the methods or acknowledgments sections of the full reports. A previous study found that 71.2% of RCTs published in 2018 reported the TRN (29). It is likely that the TRN reporting may have been improved more recently for COVID-19 trials, and the TRN is now required by many journals and preprint services (e.g., medRxiv) for clinical trials. However, the extent of the compliance of the TRN reporting in reports of registered COVID-19 trials remains unclear.

We focused on result reporting of the registered COVID-19 trials, and did not consider the outcome measures used. The sample sizes of the registered trials were planned sample sizes, and the actual sample sizes were not examined. We did not assess trials results in detail, and multiple systematic reviews are required to assess and synthesize results of relevant COVID-19 trials.

Empirical evidence revealed the existence of publication related biases where positive or favorable results are more likely to be reported than negative results (30). However, the assessment of publication bias will require a comparison of reported results and not-reported results in completed clinical trials, which is beyond the scope of the current study. Further research is required to assess the risk of publication bias in registered COVID-19 clinical trials of traditional medicine interventions.

## Conclusion

A high proportion of the COVID-19 clinical trials registered during the first 14 months of the pandemic in China and India were trials of traditional remedies. There were considerable between-country and within-country differences in date of trial registration, primary sponsors, design quality, target sample size, types of trial participants, and reporting of trial results. The probability of result reporting of COVID-19 trials of traditional medicine was lower than those of conventional medicine.

## Data availability statement

The raw data supporting the conclusions of this article will be made available by the authors, without undue reservation.

## Author contributions

FS and HF conceived the study. NZ, KP, RZ, SL, JF, HF, and FS conducted data extraction and searching of trial reports. FS conducted data analyses, prepared the draft manuscript, guarantor, and accept full responsibility for the work. JZ provided advice on result interpretation. All authors reviewed and approved the manuscript before submission.

## Footnotes

1
https://www.who.int/clinical-trials-registry-platform

## References

[1] Mahase E. Covid-19: WHO declares pandemic because of “alarming levels” of spread, severity, and inaction. *BMJ.* (2020) 368:m1036. 10.1136/bmj.m1036 32165426

[2] Xiong Y Gao M van Duijn B Choi H van Horssen F Wang M. International policies and challenges on the legalization of traditional medicine/herbal medicines in the fight against COVID-19. *Pharmacol Res.* (2021) 166:105472. 10.1016/j.phrs.2021.105472 33592272 PMC7882224

[3] Cyranoski D. China is promoting coronavirus treatments based on unproven traditional medicines. *Nature.* (2020) [Online ahead of print]. 10.1038/d41586-020-01284-x 32376938

[4] Shankar A Dubey A Saini D Prasad CP. Role of complementary and alternative medicine in prevention and treatment of COVID-19: an overhyped hope. *Chin J Integr Med.* (2020) 26:565–7. 10.1007/s11655-020-2851-y 32761336 PMC7405788

[5] World Health Organization [WHO]. *SARS: Clinical Trials on Treatment using a Combination of Traditional Chinese Medicine and Western Medicine : Report of the WHO International Expert Meeting to Review and Analyse Clinical Reports on Combination Treatment for SARS, 8-10 October 2003, Beijing, People’s Republic of China.* Geneva: World Health Organization (2004).

[6] Mahaboob Ali A Bugarcic A Naumovski N Ghildyal R. Ayurvedic formulations: potential COVID-19 therapeutics? *Phytomed Plus.* (2022) 2:100286. 10.1016/j.phyplu.2022.100286 35474908 PMC9020642

[7] Wang H Jin X Pang B Liu C Zheng W Yang F [Analysis on clinical study protocols of traditional Chinese medicine for coronavirus disease 2019]. *Zhongguo Zhong Yao Za Zhi.* (2020) 45:1232–41. 10.19540/j.cnki.cjcmm.20200220.501 32281330

[8] Glasziou P Sanders S Hoffmann T. Waste in covid-19 research. *BMJ.* (2020) 369:m1847. 10.1136/bmj.m1847 32398241

[9] Janiaud P Axfors C Hooft J Saccilotto R Agarwal A Appenzeller-Herzog C The worldwide clinical trial research response to the COVID-19 pandemic - the first 100 days. *F1000Res.* (2020) 9:1193. 10.12688/f1000research.26707.2 33082937 PMC7539080

[10] Park J Mogg R Smith G Nakimuli-Mpungu E Jehan F Rayner C How COVID-19 has fundamentally changed clinical research in global health. *Lancet Glob Health.* (2021) 9:e711–20. 10.1016/s2214-109x(20)30542-8 33865476 PMC8049590

[11] Chan A Song F Vickers A Jefferson T Dickersin K Gøtzsche PC Increasing value and reducing waste: addressing inaccessible research. *Lancet.* (2014) 383:257–66. 10.1016/s0140-6736(13)62296-5 24411650 PMC4533904

[12] R Core Team. *R: A Language and Environment for Statistical Computing (version 4.1.3).* Vienna: R Foundation for Statistical Computing (2022).

[13] Therneau T. *A Package for Survival Analysis in R. R Package Version 3.3-1.* (2022). Available online at: https://cran.r-project.org/web/packages/survival/vignettes/survival.pdf (accessed January 9, 2023).

[14] Janiaud P Axfors C Ioannidis JP Hemkens LG. Recruitment and results reporting of COVID-19 randomized clinical trials registered in the first 100 days of the pandemic. *JAMA Netw Open.* (2021) 4:e210330. 10.1001/jamanetworkopen.2021.0330 33646310 PMC7921903

[15] Salholz-Hillel M Grabitz P Pugh-Jones M Strech D DeVito NJ. Results availability and timeliness of registered COVID-19 clinical trials: interim cross-sectional results from the DIRECCT study. *BMJ Open.* (2021) 11:e053096. 10.1136/bmjopen-2021-053096 34810189 PMC8609493

[16] Mayer C Huser V. regCOVID: tracking publications of registered COVID-19 studies. *BMC Med Res Methodol.* (2022) 22:221. 10.1186/s12874-022-01703-9 35948881 PMC9364859

[17] Horby P. Why preprints are good for patients. *Nat Med.* (2022) 28:1109. 10.1038/s41591-022-01812-4 35534569

[18] Zhang T Li X Chen Y Zhao L Tian J Zhang J. Evidence mapping of 23 systematic reviews of traditional chinese medicine combined with western medicine approaches for COVID-19. *Front Pharmacol.* (2021) 12:807491. 10.3389/fphar.2021.807491 35197851 PMC8860227

[19] World Health Organization [WHO]. *WHO Expert Meeting on Evaluation of Traditional Chinese Medicine in the Treatment of COVID-19.* Geneva: World Health Organization (2022).

[20] Lee DY Li QY Liu J Efferth T. Traditional Chinese herbal medicine at the forefront battle against COVID-19: clinical experience and scientific basis. *Phytomedicine.* (2021) 80:153337. 10.1016/j.phymed.2020.153337 33221457 PMC7521884

[21] Leung EL Pan H Huang Y Fan X Wang W He F The scientific foundation of Chinese herbal medicine against COVID-19. *Engineering.* (2020) 6:1099–107. 10.1016/j.eng.2020.08.009 33520331 PMC7833648

[22] Singh RS Singh A Kaur H Batra G Sarma P Kaur H Promising traditional Indian medicinal plants for the management of novel Coronavirus disease: a systematic review. *Phytother Res.* (2021) 35:4456–84. 10.1002/ptr.7150 34132429 PMC8441711

[23] Lee CK Merriam LT Pearson JC Lipnick MS McKleroy W Kim E. Treating COVID-19: evolving approaches to evidence in a pandemic. *Cell Rep Med.* (2022) 3:100533. 10.1016/j.xcrm.2022.100533 35474746 PMC8826498

[24] Chaudhari S Somvanshi P. Methodological analysis of CTRI registered clinical trials on Ayurveda interventions for COVID-19 management. *J Ayurveda Integr Med.* (2022) [Online ahead of print]. 10.1016/j.jaim.2022.100631 35971456 PMC9365872

[25] Bhapkar V Sawant T Bhalerao S. A critical analysis of CTRI registered AYUSH studies for COVID- 19. *J Ayurveda Integr Med.* (2022) 13:100370. 10.1016/j.jaim.2020.10.012 33262559 PMC7690275

[26] Luo H Yang M Tang Q Hu X Willcox ML Liu J. Characteristics of registered clinical trials on traditional Chinese medicine for coronavirus disease 2019 (COVID-19): a scoping review. *Eur J Integr Med.* (2021) 41:101251. 10.1016/j.eujim.2020.101251 33204368 PMC7659925

[27] Tao L Zhang H Zhuo L Liu Y Qiao R Zhao Y A scientificity and feasibility evaluation of COVID-19 clinical studies registered in China. *Ann Transl Med.* (2020) 8:817. 10.21037/atm-20-2943 32793662 PMC7396233

[28] Mullard A. RECOVERY 1 year on: a rare success in the COVID-19 clinical trial landscape. *Nat Rev Drug Discov.* (2021) 20:336–7. 10.1038/d41573-021-00068-w 33864035

[29] Al-Durra M Nolan R Seto E Cafazzo J. Prospective registration and reporting of trial number in randomised clinical trials: global cross sectional study of the adoption of ICMJE and Declaration of Helsinki recommendations. *BMJ.* (2020) 369:m982. 10.1136/bmj.m982 32291261 PMC7190012

[30] Song F Parekh S Hooper L Loke Y Ryder J Sutton A Dissemination and publication of research findings: an updated review of related biases. *Health Technol Assess.* (2010) 14:1–193. 10.3310/hta14080 20181324

