# Supplementary material for: Characteristics and result reporting of registered COVID-19 clinical trials of Chinese and Indian traditional medicine: A comparative analysis
Source: Front Med (Lausanne). 2023 Feb 17;10:1118269. doi: 10.3389/fmed.2023.1118269 (PMC9981796; doi:10.3389/fmed.2023.1118269)
Supplement: Supplementary file 1 [file Data_Sheet_1.docx]

**Supplementary material**

Characteristics and result reporting of registered COVID-19 clinical trials of Chinese and Indian traditional medicines: a comparative analysis

**Supplementary figure 1:**

### Dates of newly registered COVID-19 trials and reported daily new cases in China, India, and other countries.

Data sources: Registered COVID-19 clinical trials from WHO ICTRP databases and the number of daily reported COVID-19 cases from WHO Coronavirus (COVID-19) Dashboard (https://covid19.who.int/). Included trials were registered before 1 February 2021, and reported daily new cases were by 31 October 2021.

**Supplementary figure 2:**

### The distribution of target participants by trial group.

Notes: “severe” included cases from moderate to severe or critical severity; “mixed” refers to trials that included any suspected or confirmed COVID-19 cases, or did not specify disease severity; “nonsevere” included asymptomatic, mild to moderate severity; “recovery” refers to individuals who were recovering or recovered from COVID-19; and “other” included healthy volunteers, individuals at high risk of being infected, and other individuals without COVID-19.

**Supplementary figure 3:**

### The proportion of trials recruiting different types of participants by sample size categories.

Note: Pearson's Chi-squared test with simulated p-value: X-squared = 205.89, df = NA, p-value = 0.0001666


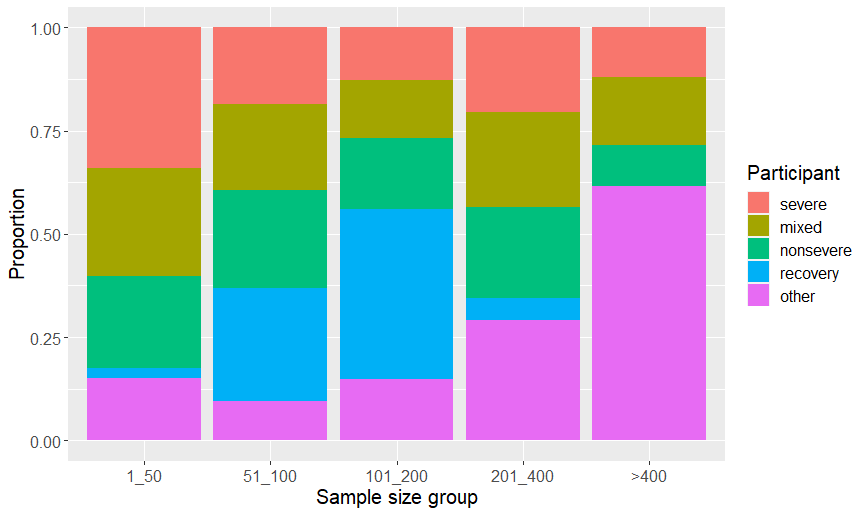


**Supplementary figure 4:**

### Median days (IQR) from trial onset to result reporting for registered COVID-19 trials that reported results by 20 April 2022


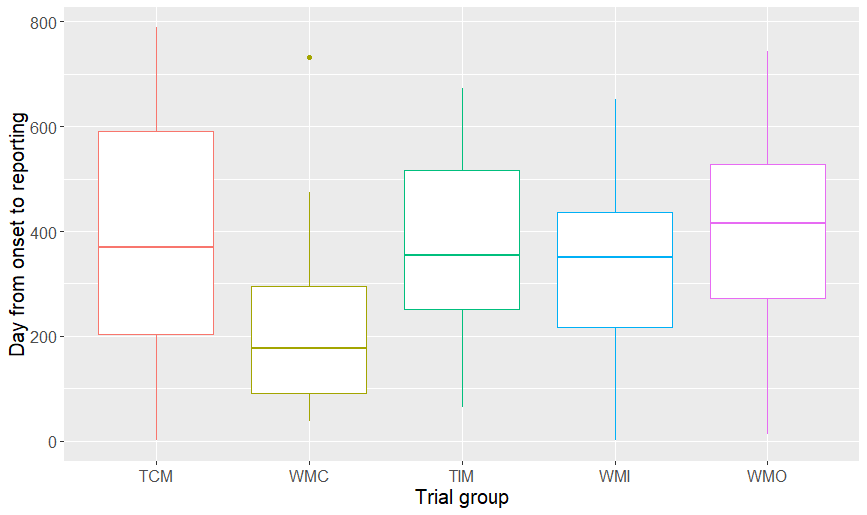


| **Trial group** | **Median** | **IQR** |
| --- | --- | --- |
| TCM | 370.0 | (203.0 – 590.0) |
| WMC | 176.5 | (91.3 – 295.3) |
| TIM | 354.5 | (251.2 - 516.2) |
| WMI | 349.5 | (216.8 – 437.0) |
| WMO | 416.0 | (273.0 – 528.0) |

Kruskal-Wallis chi-squared = 52.404, df = 4, p-value = 1.135e-10
